# Quality indicators of telemedical care offshore—a scoping review

**DOI:** 10.1186/s12913-021-07303-5

**Published:** 2021-12-02

**Authors:** Michael Hellfritz, Alexander Waschkau, Jost Steinhäuser

**Affiliations:** Institute of Family Medicine, University Medical Center Schleswig-Holstein, Campus Lübeck, Ratzeburger Allee 160, 23562 Lübeck, Germany

**Keywords:** Offshore, Oil and gas industry, Offshore wind energy, Telemedicine, Medical care, Quality, Quality Indicator, Scoping review

## Abstract

**Background:**

Offshore industries operate all around the world in diverse and remote environments. The use of telemedicine to ensure up-to-date medical care for thousands of people offshore has been common practice for decades. Thus, in this setting, extensive experiences with this type of health care delivery have already been gathered, while in other settings this is just beginning. However, the quality of telemedical care on offshore installations is rarely reported yet. The objective of this review was to explore published literature with regards to the following questions: Have any Quality Indicators (QIs) been published for measuring the quality of telemedical care on offshore installations or are there identifiable items that could be used as such QIs?

**Methods:**

We conducted a comprehensive Scoping Review (PRISMA-ScR) of the published literature using the databases MEDLINE, Cochrane Library, Web of Science (Core Collection), and Google Scholar. Search results were read and QIs or findings from which QIs could be derived were classified according to the dimensions of quality established by Donabedian (structure, process, or outcome QIs).

**Results:**

The search returned 10,236 non-recurring articles, 45 of which were full-text screened and 15 of which were included in this review. Types of publications were heterogenous. No QIs for the quality of telemedical care offshore have been published yet. Findings that could be the basis for QIs focused on structure quality (11 QIs) followed by process quality (11 QIs), while outcome quality was less common (1 QI).

**Conclusion:**

Currently, although years of experience with telemedical care on offshore installations exist, there is a paucity of research on a solid data base regarding the quality of telemedical care offshore. The authors derived a list of 24 possible QIs from the findings of the publications for further validation. This could be the basis for implementation and definition of QIs in this and in similar remote settings.

**Supplementary Information:**

The online version contains supplementary material available at 10.1186/s12913-021-07303-5.

## Background

Offshore wind and offshore oil and gas industries operate all around the world. It is estimated that, in 2008, at any particular time, 15–28,000 people lived and worked offshore in and on the entire North Sea alone [1]. The United Kingdom (UK) Oil and Gas Industry Association counted almost 50,000 workers travelling offshore in the UK sector in 2017, with over 23,000 of them spending at least 100 nights offshore [2].

Offshore installations can vary greatly in size, manning, and function. While some oil drilling rigs permanently accommodate up to several hundred people, offshore wind structures may only be visited irregularly by small teams of three people. Legal requirements for the provision of medical care in the offshore oil and gas industry vary internationally [3, 4]. Up to now, the much younger offshore wind industry has relied heavily on the self-imposed standards of the oil and gas industry.

According to industries Health, Safety & Environment standards, a medical professional—usually called ‘Medic’—should be stationed at any major installation. ‘Medic’ generally refers to a medical professional (e.g. paramedic, emergency medical technician, nurse, medical doctor) with experience in emergency medical care and with advanced life support skills [3, 5, 6].

To support the provision of medical care offshore, telemedicine has been common practice for decades [6]. Initially referring to telephone and radio communication, nowadays telemedical solutions often include live videoconferencing, transmission of advanced subjective and objective medical information and a coordinated specialized medical team onshore [1, 6, 7].

Epidemiology patterns of offshore medical emergencies and reasons for evacuations have changed over the past five decades. In the 1970s, substantially more injuries than illnesses occurred offshore. In 1985, out of 743 consultations on offshore installations dealt with by the medical support doctors onshore, 29% were successfully treated only with advice over radio to the offshore medical personnel. In the 1990s, illnesses made up more than 55% of the reasons for evacuation, with musculoskeletal, dental, and respiratory illnesses being the most common, and cardiovascular, skin, mental, and genitourinary illnesses being the least common reasons for evacuation. Most commonly evacuated injuries were sprains, strains, and fractures [3].

Thus, in offshore settings, extensive experiences with telemedical care have been gathered in thousands of consultations over decades [8]. However, although existing literature partly covers aspects of quality, there is only a very limited number of publications focusing on the quality of telemedical care on offshore installations.

The use of telemedicine in other areas has increased over the past years [9]. Organizational issues have not been adequately examined yet [10]. Telemedical care projects in other settings, e.g., rural and remote areas, could benefit from the experiences and research findings of offshore telemedical care.

Donabedian established a comprehensive approach for the assessment and evaluation of the quality of medical care, introducing three dimensions of quality: structure, process, and outcome quality. Structure quality describes the material, personnel, and administrative conditions that medical care is delivered in. Process quality maps the immediate interaction between the physician, the medical assistants, the administrators, and the patient. And outcome quality describes the outcome of the medical condition of the patient. Analyzing these dimensions of quality of care can result in measurable quality indicators (QIs) [11]. Such QIs are essential for documenting and improving the quality of medical care [12].

The objective of the present study was to explore the published literature with regards to the following questions: Which QIs describing the quality of telemedical care on offshore structures have been published? Are there any items in existing publications that could be used as a basis for such QIs?

## Methods

On August 11th, 2021, a comprehensive Scoping Review was carried out, using the databases and meta-databases MEDLINE, Cochrane Library, and Web of Science (Core Collection). It followed the guidelines of the PRISMA Extensions for Scoping Reviews (PRISMA-ScR) [13] (see Additional file 1), and was not registered prior to the search or publication. It was supplemented by a search for grey literature with Google Scholar.

### Search strategy

To ensure a maximum spectrum of relevant results, we developed a comprehensive search strategy including MeSh terms. Relevant MeSh terms were identified in an analysis of the MeSh term database of the National Library of Medicine [14]. Additional search terms were identified by screening the author-supplied keywords on PubMed to ensure that the latest articles which had not yet been indexed were also included. The wide scope of the review question and the previously experienced lack of published literature and evidence on the subject led us to search all fields of the database-entries, not only title and abstracts. In addition, we searched in common language to identify as many relevant papers as possible and especially publications not yet indexed. The search strategy included the primary search terms “telemedicine”, “remote consultation”, “oceans and seas”, “naval medicine”, “emergencies”, “emergency medicine”, “emergency medical services”, “rescue work”, “first aid”, “accidents”, “oil and gas industry”, “fossil fuels”, and “oil and gas fields”, as well as the keywords “telemedicine”, “telehealth”, “remote consultation”, “teleconsultation”, “offshore”, “oceans”, “nautical medicine”, “emergency”, “emergency medicine”, “emergency medical service”, “emergency health service”, “emergency care”, “rescue work”, “first aid”, “accidents”, “petroleum industry”, “fossil fuel”, and “oil and gas fields”. Key words concerning the oil and gas industry were deliberately included in the search because this industry has been the main drive for innovation on offshore installations over the past decades.

Additionally, a search for grey literature was carried out using the main search terms “telemedicine”, “offshore wind”, “offshore oil and gas” and “quality”. For this search, Google Scholar was used in private mode to ensure replicability of result.

Furthermore, the reference lists of included publications were cross-checked for potentially relevant articles.

The complete search strategy is available in the supplementary material (see Additional file 2).

The results of the searches were imported into the online review tool Covidence [15] which was used in the subsequent process.

### Screening procedure, inclusion and exclusion criteria

After removal of duplicates, all publications were included in the subsequent review process. All titles and abstracts were screened for potential relevancy. Potentially relevant papers were assessed for eligibility based on inclusion and exclusion criteria following the PCC-approach (population, concept, context) [16]. The PCC elements are listed in Table 1. The screening process has been conducted by two reviewers (AW, MH) separately. Consensus meetings were held with an experienced third reviewer (JS) as needed.

**Table 1:** PCC elements for inclusion and exclusion criteria

PCC-element	Definition
Population	Medical expert users of telemedicine
concept	QIs describing the quality of telemedical care
	published items, that could be the basis for QIs
context	offshore oil and gas or offshore wind industry

Publications were included if they met the following criteria:

- Focus on medical expert users of telemedicine
- QIs describing telemedical care or items that could be used as such QIs were identifiable
- Operation of telemedicine in offshore wind or offshore oil and gas industry

Publications were excluded for the following reasons only:

- No focus on telemedicine
- No focus on offshore installations
- No full text available
- Not addressing QIs or items that QIs could be derived from

There were no restrictions regarding language and type of study. We included all relevant publications regardless of date of publication.

All publications included in this review were read systematically, and relevant information was extracted. Findings were classified according to the dimensions of quality established by Donabedian [11].

## Results

Altogether 14,012 publications were identified. The search for grey literature yielded 352 papers.

After removal of duplicates, a total of 10,236 publications were included in the screening process. Out of these, 49 potentially relevant papers were assessed for eligibility based on inclusion and exclusion criteria. No explicit QIs regarding the quality of telemedical care offshore were found. However, 15 publications were included in this Scoping Review as they contained findings, from which QIs could be derived.

**Fig. 1:**
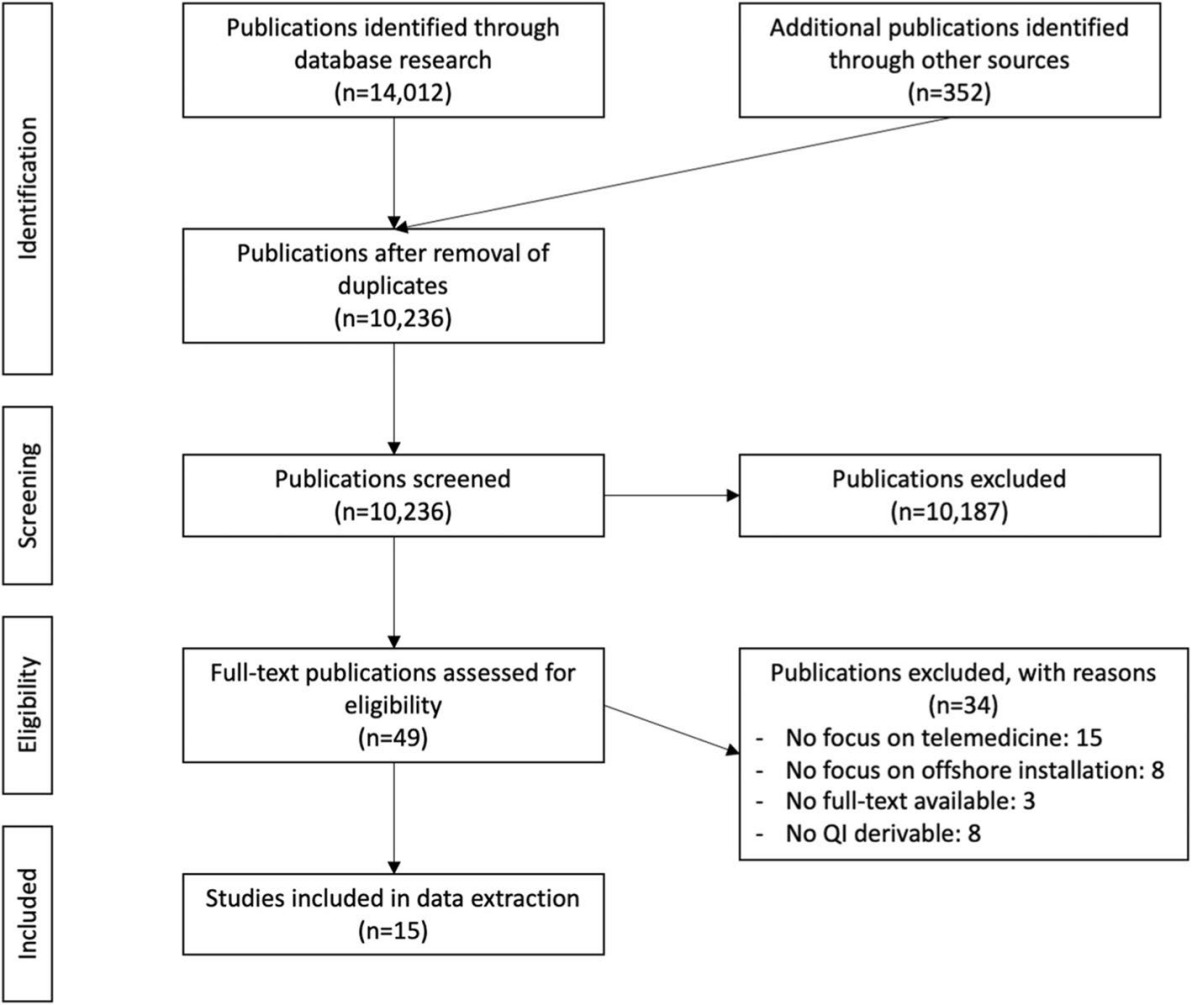
PRISMA Flow Chart

The identification and selection process is visualized in the PRISMA Flow Chart in Fig. 1.

The 15 publications included eight journal articles [3, 4, 17–22], five conference reports [6, 23–26], one doctoral thesis [27], and one chapter of a book [28]. Table 2 shows a summary of the publications.

**Table 2:** Summary of publications in chronological order

Authors	Year	Title	Country	Type of research	Aim
Mika F. et al. [25]	2007	Development of a Post-Graduate Qualification Course in Telemedicine and Telepharmacy for Physicians in Offshore Oil and Gas Industry	USA	Conference proceeding	Background information on a qualification course in telemedicine and telepharmacy for offshore oil and gas installations.
Webster K. et al. [19]	2008	A low-cost decision support network for electrocardiograph transmission from oil rigs in the North Sea	UK	Test report	Analysis of feasibility and effect of a low-cost ECG-telemedicine device on offshore oil installations.
Ponsonby W. et al. [3]	2009	Offshore industry: medical emergency response in the offshore oil and gas industry	Netherlands	Literature review, Summary of current practice	Literature review to define challenges of Medical Emergency Response, gives examples of legal requirements, and summarize current practices in the oil and gas industry.
Fernandes A. et al. [23]	2014	Development of Telemedicine in Oil & Gas through the Capabilities Approach	Norway	Conference proceeding	Transfer of the Capability Approach within the concept of Integrated Operation onto the implementation and development of offshore telemedicine.
Pelat F. et al. [26]	2014	Learning & benefits of well-defined and well-structured topside medical support in the offshore drilling industry based on 10 years of global experience with a large offshore drilling contractor	USA	Conference proceeding	Description and review of structuring “Topside Medical Support” on offshore drilling installations.
Thorvik K. et al. [24]	2014	The future of telemedicine in Oil & Gas	Norway	Conference Proceedings	Report of a telemedicine prototype study to improve telecare offshore.
Dubrowski A [17].	2015	Simulation as a suitable education approach for medical training in marine and offshore industries: theoretical underpinning	Canada	Theoretical underpinning	Theoretical rationale for simulation as a concept for medical training for offshore and marine medical practitioners.
Evjemo T. et al. [6]	2015	Telemedicine in Oil and Gas: Current status and potential improvements	Norway	Mixed method qualitative analysis	Identification and generalization of good practices, central challenges, and lessons learned of current telemedicine solutions in petroleum industry.
Carius C. et al. [28]	2016	SOS auf Offshore-Plattform Sieben	Germany	Project report	Report of a telemedical emergency care concept for offshore wind turbines.
Loddo M [4].	2017	The maritime qualified emergency teledoctor in offshore areas	Germany	Magazine article, Statement	Progress report and lessons learned of an offshore medical control center.
Landgraf P. et al. [18]	2019	﻿Does Telemedical Support of First Responders Improve Guideline Adherence in an Offshore Emergency Scenario? A Simulator-Based Prospective Study	Germany	Prospective study	Simulator-based prospective study of quality of emergency response by telemedically supported non-professionals in comparison to medical professionals.
Landgraf P [27].	2020	Effects of Telemedical Support on Quality of Emergency Information Retrieval Considering Offshore Wind Power Infrastructure	Germany	Doctoral thesis	Simulator-based prospective study of quality of emergency response by telemedically supported non-professionals in comparison to medical professionals.
Huzaini A. et al. [21]	2020	﻿Exploring of Offshore Medical Emergency Response System Challenges in Oil and Gas Environment	Malaysia	Qualitative interviews	Exploration of challenges in the offshore medical emergency response system
﻿Vatsvåg V. et al. [20]	2020	Offshore telementored ultrasound: a quality assessment study	Norway	Quality assessment study	Assessment of feasibility and quality of telementored ultrasound in offshore setting
Mastella G. et al. [22]	2021	Offshore telemedicine emergency service: a 1-year experience	Germany	pilot test project	Determination if telemedical emergency care offshore is possible in the North Sea

The types of publication were heterogenous, varying from prospective studies [18, 20, 22, 27], qualitative analyses [6, 21], a literature review [3], and a theoretical underpinning [17] to retrospective experience reports, partly with no scientific data base [4, 19, 23–26, 28]. Seven articles addressed quality of telemedical care offshore [6, 20–24, 26], five articles covered areas relevant for the topic scientifically [17–19, 25, 27], and three articles discussed the topic without any data base [3, 4, 28]. Seven of the fifteen publications were identified through grey literature research and reference cross check instead of medical database inquiry [19, 21, 23–26, 28].

### Structure quality

Ten of the fifteen publications dealt with topics relevant for structure quality such as technical equipment, coordination structures, and best approaches for the development of quality of telemedical care offshore [3, 4, 6, 19, 21–25, 28].

Five publications dealt with technical equipment for telemedicine. Five of them stressed the importance of reliably sharing real time objective information via audio/video link, phone, fax, or email [3, 6, 19, 21, 24], and one described the technology used [28]. Three publications favored an easy to use “plug-and-play”- or “one button push” solution over complex medical and communication equipment. Complex equipment may lead to a distraction from the patient and may therefore negatively affect treatment [22, 24, 28].

### Process quality

Ten of the fifteen articles covered issues concerning process quality, such as the importance of communication processes and human resources that ensure quality [3, 4, 17–21, 24–26]. Two articles discussed qualifications of both, the teleconsultant physicians onshore and the medical personnel offshore [4, 25]. Two other articles dealt with the effects of contextualized simulation training for medical personnel offshore [17, 18]. One article stressed the importance of adjusting to end-user acceptance when implementing telemedical solutions [22].

### Outcome quality

Only one of the fourteen publications addressed outcome quality. The authors reported that expert ECG interpretation onshore and advice on the management of chest pain offshore affects the rate of evacuation [19].

In Table 3, findings, from which QIs could be derived are summarized.

**Table 3:** Findings eligible for QIs and consequent QIs

Dimensions in Donabedian’s Framework	Findings eligible as future quality indicator	Derived QIs	
Structure	Existence of reliable, continuous, and transmittable equipment-based, “plug-and-play” monitoring of vital parameters offshore, including 12-lead ECG [4, 21, 22, 24, 28].	1	Reliably transmittable continuous equipment-based monitoring of vital parameters offshore available within 15 min / all medical cases. ^a^
		2	“Plug-and-Play” telemedical equipment available within 15 min / all telemedical equipment. ^a^
		3	12-lead ECG/all ECG-machines.
	Existence of reliable, high-quality videoconferencing system [24].	4	High-quality video conferencing systems available within 15 min / all medical cases. ^a^
		5	“Plug-and-Play” video conferencing system available within 15 min / all medical cases. ^a^
	Development of telemedical care concepts follows a systemic approach [6, 21, 23].	6	Existence of telemedical care concept guidelines / offshore installation.
	Telemedicine is adjusted to the available bandwidth [6].	7	Number of automatically bandwidth-adjusting telemedical equipment / all telemedical equipment.
	Subjective and objective medical data as well as patient’s history is accessible through telemedical equipment in real time for several onshore and offshore experts simultaneously and without intermediary [6, 22, 24].	8	Number of telemedically accessible Electronic Health Records (EHR) / number of staff offshore.
	Major hospital is available 24/7 for specialist consulting. Additional specializations are accessible at all times [4].	9	Number of hours a major hospital is available for synchronous specialist consulting / 24 h
	Qualification of a teleconsultant physician onshore and medical personnel offshore needs to be defined [4, 25].	10	Teleconsultant physicians according to predefined qualifications / all active teleconsultant physicians.
		11	Offshore medical personnel according to predefined qualifications / all active offshore medical personnel.
Process	Personal identification of involved personnel is documented and accessible [6].	12	Number of personnel with documented and accessible professional identification / all involved personnel.
	Communication between and equipment at locations onshore and offshore is standardized [6, 26].	13	Proportion of equipment offshore and onshore that is standardized within the operation / all used equipment.
		14	Number of telemedical communication processes between offshore and onshore that are standardized within the operation / all occurring communication processes.
	Medical protocols and procedures for the most common cases are defined [20, 26].	15	Most common medical cases, which have defined protocols / 20 most common cases.
	Development of telemedical care concepts is communicated systematically to all relevant parties [6, 21, 23].	16	Number of changes to telemedical care concepts, that were communicated systematically to all relevant parties / all changes to telemedical care concepts.
	New workflow processes are aligned with tacit knowledge, experience, and preferred improvements of involved staff [6].	17	Number of new workflow processes that follow participatory design / all new workflows introduced within the last 12 months.
	New telemedical solutions are aligned with end-user acceptance [22].	18	Number of telemedical solutions aligned with end-user acceptance/ all telemedical solutions in place.
	Personnel using telemedicine has completed a structured, contextualized telemedical training [17, 18, 25, 27].	19	Number of personnel using telemedicine, that has completed a structured contextualized telemedical training / all personnel using telemedicine.
	Medical experts are directly included in the decision-making process offshore [6].	20	Number of times, medical experts were directly included in the decision-making process offshore / all consultations.
	Decisions on telemedical issues are medically driven only [21].	21	Purely medically driven decisions on telemedical issues / all decisions on telemedical issues.
	In an emergency, medical personnel is assisted offshore in non-medical tasks to be able to focus on medical treatment [21].	22	Number of telemedical cases, in which medical personnel is assisted by non-medical staff / all telemedical cases.
	Most common medical cases are followed-up to improve procedures and design trainings that improve quality [6].	23	Most common cases, that were followed up to improve procedures and design trainings that improve quality / 20 most common cases.
Outcome	Analysis of evacuations in relation to total number of consultations [19].	24	Consultations followed by evacuation / all consultations.

^a^Medical response time is derived from legal requirements for maximum medical response times onshore in Germany

## Discussion

We could not identify any explicit QIs for telemedicine on offshore structures in the reviewed literature. However, there were relevant findings from which 24 possible future QIs could be derived.

Six out of the fifteen publications identified were case or experience reports [4, 23–26, 28]. Therefore, studies resulting in a higher level of evidence are needed.

Minimum standards in health care legislation for offshore settings vary greatly and lack international standardization [3, 4]. Oil and Gas UK (OGUK) and the International Petroleum Industry Environmental Conservation Association (IPIECA) both have issued guidelines on medical equipment and facilities for their offshore installations [5, 29, 30] but no specific recommendations for the use of telemedicine could be identified. The general approach and standardization for health and safety in the offshore wind industry should be systematic and has not yet reached the same level as in the offshore oil and gas industry [31]. The European Union has announced to standardize and publish health, safety and environment recommendations for the industry in the future [32] but so far, no official plans for the implementation of QIs have been published.

According to one author, there are several possible designated uses for telemedicine offshore [3]:

- First medical opinion: to interpret medical reports, e. g. X-ray or electrocardiogram (ECG).
- Second medical opinion: to define whether the case is an emergency or to evaluate the level of urgency and the need for evacuation or for advice regarding the treatment before and during evacuation.
- Shared clinical management: management of patients with a temporary diagnosis or of complex cases by a “coordination unit” located at a specialist referral center assures a “single case – single connection – multiple response” model.
- Management of chronic diseases offshore.

However, the designated use is not standardized within the industry and there is no exact definition in the analyzed literature.

QIs 12 to 15 aim to facilitate intuitive cooperation in varying scenarios where personnel is mostly unfamiliar, far away, and has different levels of expertise. Several authors reported that the standardization of equipment, communication, and procedures have proven to be effective in this facilitation [24, 26]. While this finding seems plausible, no research regarding its necessity or the appropriate extent of standardization in the offshore telemedicine could be identified. The derived QIs therefore represent the beginning of a process that will result in more differentiated QIs. Future research steps should include development of more precise QIs, using e. g. the RAND UCLA Appropriateness method [33], including findings and QIs from this review. Resulting QIs should then be validated [34].

QI 10, 11, 15 and 23 aim to improve quality of medical care by ensuring that personnel is trained and procedures are fit for the medical cases that are to be expected. Their efficiency though is only as good as its underlying research. There was no close analysis identified regarding the spectrum of medical cases offshore. For the oil and gas industry, the most common reasons for consultation and evacuation have been analyzed [8, 35, 36] but for the younger offshore wind industry, there is only little equivalent research available [37–39]. But situations may differ significantly. The spectrum of medical cases and the most common medical issues need to be further analyzed, and resulting appropriate qualifications need to be discussed and validated in future research. Two articles report on experiences with qualifications of consultant physician onshore and medical personnel offshore [4, 25]. It seems necessary, that further structured research is undertaken about necessary qualifications. This research, however, must take into consideration an analysis of the spectrum of medical cases and the most common medical issues that involved personnel is confronted with. Resulting qualifications then should be transferred into a curriculum and be validated.

None of the findings identified in this review have dealt with the needs or the experience of the patient. Future research on telemedical care offshore needs to include this aspect as well, and resulting QIs need to be included in a set of QIs.

Findings from this review may be relevant for other areas of use of telemedicine too. E. g. QIs 1, 2 and 4–8 aim to promote a reliable connection, with intuitive equipment and a smooth process of consultation. QIs 9–11, 15, 19 and 20 aim to measure determinants of quality of the telemedical consultation itself and QIs 16, 17 and 23 aim to measure determinants of its improvement. This could be desirable for other scenarios as well. The defining aspects of QIs could, to some extent, be applicable. Therefore, the derived QIs may very well be not only the starting point of development of QIs for offshore telemedical care but may also feed into the definition and implementation of QIs for other scenarios in which medical personnel seeks senior medical expertise via synchronous virtual consultation.

### Limitations

Our Scoping Review may be limited due to publications we could not identify because they are listed in other databases than the ones analyzed. Seven out of the fourteen publications included in the review were found in the grey literature search via Google Scholar and the reference cross check [19, 21, 23–26, 28] and not in the medical databases that we searched in. Researchers in the area of quality improvement of telemedical care offshore may not be medically associated or may publish in other databases than medical researchers.

Findings that QIs could be derived from mainly originate from literature concerning the offshore oil and gas industry. Several articles have been published or financed by oil and gas companies, their employees, or associated societies, so there may be a bias in findings [3, 23–26]. Some findings originate from authors with business interests in the field so their findings may be biased as well [4].

Situations offshore may differ significantly. While the offshore oil and gas industry operates in stationary and large groups of up to several hundred people, offshore wind workers are sometimes deployed in flexible groups of three workers only. The field of telemedicine offshore therefore needs to be differentiated, principles of operation and quality are only partially transferable from one scenario to another [4, 40, 41].

Fast-developing technology may also lead to outdated findings that need to be evaluated carefully. Some articles report technical requirements that are already rather outdated compared to the medical and technical equipment used nowadays on ships and offshore installations, e. g. in the German Bight [3, 19]. The findings of this review are not intended to be generalized to the whole field of telemedicine, rather, they should be an impetus to address the issue of quality of telemedical care more firmly.

## Conclusion

The quality of telemedical care offshore is mainly discussed without a solid data base in scientific publications, and no set of QIs or single QIs have been published yet. Overall, there is little evidence available and more research, using advanced methods, needs to be done. QIs need to be developed, validated, and evaluated. This review provides a first set of 24 QIs to start this process. These QIs may also be a starting point for the development of QIs in other areas of use of telemedicine.

## Supplementary Information

**Additional file 1.** Preferred Reporting Items for Systematic reviews and Meta-Analyses extension for Scoping Reviews (PRISMA-ScR) Checklist.

**Additional file 2.** Complete search strategy.

## Data Availability

All data generated or analysed during this study are included in this published article and its supplementary information files.

## Abbreviations

ECG: Electrocardiogram
EHR: Electronic Health Record
IPIECA: International Petroleum Industry Environmental Conservation Association
OGUK: Oil and Gas UK
QI: Quality indicator
UK: United Kingdom
