# Supplementary material for: Quality indicators of telemedical care offshore—a scoping review
Source: BMC Health Serv Res. 2021 Dec 2;21:1290. doi: 10.1186/s12913-021-07303-5 (PMC8638379; doi:10.1186/s12913-021-07303-5)
Supplement: Supplementary file 2 — Additional file 2. Complete search strategy. [file 12913_2021_7303_MOESM2_ESM.docx]

**Additional file 2**

1. **Search-strategy**

| **Mesh-terms** | **Search-terms - Keywords** |
| --- | --- |
| telemedicine | telemedicine |
| remote consultation | telehealth |
| Oceans and Seas | remote consultation |
| naval medicine | teleconsultation* |
| emergencies | oceans |
| emergency medicine | nautical medicine |
| emergency medical services | emergency |
| rescue work | emergency medicine |
| first aid | emergency medical service |
| accidents | emergency health service |
| oil and gas industry | emergency care |
| fossil fuels | rescue work |
| oil and gas fields | first aid |
|  | accidents |
|  | petroleum industry |
|  | fossil fuel |
|  | oil and gas fields |

**Searchterm MEDLINE 11.08.2021:**

(((((telemedicine) OR telehealth) OR remote consultation) OR teleconsultation*)) AND ((((((oceans) OR nautical medicine) OR offshore*)) OR ((((((((emergency) OR emergency medicine) OR emergency medical service) OR emergency health service) OR emergency care) OR rescue work) OR first aid) OR accidents)) OR (((petroleum industry) OR fossil fuel) OR (oil and gas fields)))

**Searchterm Grey Literature 11.08.2021**

(telemedicine AND “offshore wind” AND quality)

**Searchterm Grey Literature 11.08.2021**

(telemedicine AND "offshore oil and gas" AND quality)
